# System for the Acquisition and Monitoring of Energy Consumption in the Context of Industry 4.0

**DOI:** 10.3390/s24196302

**Published:** 2024-09-29

**Authors:** Tudor Covrig, Alexandru Ciobotaru, Pavel-Alexandru Bejan, Lucian Farmathy-Pop, Stelian Oltean, Cristian-Dragoș Dumitru, Liviu-Cristian Miclea

**Affiliations:** 1 Automation Department, Tehnical University of Cluj-Napoca, 400027 Cluj-Napoca, Romania or tudor.covrig@umfst.ro (T.C.); alexandru.ciobotaru@campus.utcluj.ro (A.C.); alex_bejan2003@yahoo.com (P.-A.B.); lucian.farmathy@gmail.com (L.F.-P.); 2 Department of Electrical Engineering and Computers, University of Medicine, Pharmacy, Sciences and Technology of Târgu Mureș, 540088 Târgu Mureș, Romania; stelian.oltean@umfst.ro (S.O.); cristian.dumitru@umfst.ro (C.-D.D.)

**Keywords:** Node-RED, IoT, modbus, Sentron PAC 3200, augmented reality, Industry 4.0

## Abstract

This paper examines methodologies for monitoring energy consumption and operational parameters of an industrial assembly line, with a particular emphasis on the utilization of virtual tools, augmented reality, and real-time data visualization. The research examines the integration of an interface and the implementation of a data acquisition system, employing augmented reality to facilitate interaction with the collected data. The proposed system employs the Node-RED interface to facilitate the establishment of connections between constituent elements. The data is stored in a database, which provides support for decision-making in the analysis of energy consumption and operational parameters of the industrial assembly line. The results demonstrate that the solution is effective in enhancing energy resource management, identifying inefficiencies, and optimizing the performance of industrial equipment. The findings indicate that the implementation of such a system can markedly enhance the industrial process.

## 1. Introduction

The field of industry is one that is in a state of constant evolution, with notable shifts occurring in the manner in which data is managed and organized. The advent of digital technologies has facilitated the collection and visualization of data, thereby streamlining decision-making processes and optimizing monitored processes. Consequently, the acquisition and visualization of data are indispensable for the provision of diverse forms of information. Industry 4.0 represents a transition and evolution towards the latest technological developments, which have the potential to significantly enhance industrial processes. As outlined in the paper [1], the evolution of industry can be divided into four distinct stages. The initial stage is characterized by the utilization of steam and water-powered systems. The second stage marks the advent of electricity as the primary source of energy. The third stage is distinguished by the utilization of digital logic and automated technological processes. The fourth stage pertains to the incorporation and utilization of digital environments in industrial processes. The technologies that define technological evolution include IoT (Internet of Things) [2], artificial intelligence, augmented reality, sensors, and network analyzers. A comprehensive overview of these technical solutions can be found in the referenced paper. Furthermore, operational efficiency is enhanced through the identification and elimination of deficiencies in production processes, thereby enabling decisions to be made based on real-time data. The acquisition and transmission of data are conducted via communication protocols, including S7comm [3], OPC (Open Platform Communications) [4], and Modbus [5]. It is imperative that this data be presented in a clear and readily comprehensible manner, as this will facilitate the rapid identification of potential issues. To this end, a variety of interfaces may be constructed using web technologies, SCADA (Supervisory Control and Data Acquisition) systems, and augmented reality [6]. The prevailing tendency within the industry is to advance towards the subsequent phase, which is exemplified by Industry 5.0. The defining concept of this phase is the integration of human and machine collaboration. This represents a significant advancement in industrial evolution, particularly in the domains of storage and logistics operations [7].

The objective of this paper is to present a methodology for the design and implementation of a solution for the automatic monitoring and analysis of operational data from an assembly line. The solution entails the utilization of a Sentron PAC 3200 2.1.0 network analyzer, through which energy consumption data is accumulated. By leveraging augmented reality, an algorithm is devised to facilitate the automatic interpretation of the data and its subsequent display in a dashboard. The interconnection of elements is facilitated by the Node-RED 3.1 interface, which enables the transfer of data. The acquisition and processing of data are conducted automatically. The authors are unaware of any previous attempts to adopt this approach in the specialized literature. The article is structured as follows: The second section presents an overview of the current state of the field, the third section outlines the design of the application, the fourth section describes its implementation, the fifth section presents the results obtained, and the sixth section presents a discussion of these results. The final section presents the conclusions of the paper.

## 2. The State-of-the-Art

The concept of Industry 4.0 describes the industrial sector and the characteristics that define this transformation. It represents a novel concept that entails the integration of diverse technologies. The principal technologies that define this concept are the IoT, artificial intelligence (AI), big data, augmented reality (AR), and robotics. These technologies are employed with the objective of enhancing industrial processes [8]. The current trend is the evolution from Industry 4.0 to Industry 5.0. The technologies that define and support this concept include artificial intelligence, which facilitates enhanced human-machine interaction [9]. This is also underscored in [10], which elucidates the impact of human-machine interaction on the Industry 4.0 concept. The concepts presented in this work pertain to the enhancement of the interaction between users and technological equipment. The aforementioned technologies facilitate the collection of diverse data, which can then be utilized to inform decision-making processes. One of the principal aspects of data collection and management is the IoT technology. As previously outlined in [11,12,13], the IoT represents a network of interconnected devices that are capable of communicating and exchanging data using the MQTT (Message Queue Telemetry Transport) communication protocol. This configuration constitutes a network of numerous intelligent devices, each capable of exchanging information with one another. This network may be regarded as a global entity, facilitating interaction between humans and equipment. The data collected may be interfaced with LabVIEW 3.1 software. An alternative approach to data collection is based on the use of sensors and associated technologies that facilitate the transfer of information in real time. A data acquisition method utilizing multiple sensors is outlined in [14]. To gain access to the data obtained, the Node-RED 3.1 interface may be utilized. As stated in [15], this interface enables users to link disparate devices in order to obtain data regarding their operation via the utilization of assorted communication protocols. As demonstrated in [16], the Node-RED 3.1 interface offers significant advantages, including more efficient resource utilization, faster solution implementation, and a constant evolution of the application architecture. The objective is to develop an interface that enables the deployment of an IoT application on an OpenStack-based platform, thereby ensuring comprehensive compatibility.

In addition to sensors utilized for data collection, other industrial equipment can be employed to ascertain the operational characteristics of a system. One such device is the Sentron PAC 3200 2.1.0 [17]. The aforementioned apparatus enables the instantaneous acquisition of data pertaining to energy consumption. Another technology that can be employed in conjunction with the Sentron PAC 3200 2.1.0 is the SCADA system, as detailed in [18,19]. For further information on SCADA technology, please refer to the following sources: [20,21]. Such systems facilitate the monitoring and supervision of technological processes, thereby enabling the identification of various operational anomalies based on the data obtained. Such systems may also be employed for the control of disparate industrial process simulation applications. One such industrial process simulator is the EasyPLC system. The software permits users to construct a variety of simulations through which disparate operational scenarios may be evaluated [22].

Another technology that can be utilized for the monitoring of parameters and data is augmented reality. As posited by [23], augmented reality represents a convergence of virtual and tangible environments, enabling users to engage with virtual entities while simultaneously perceiving the surrounding physical reality. The objective of these technologies is to enhance the user experience by providing supplementary information or interactivity in the context of the real world. In this manner, augmented reality enables a more profound level of interaction and comprehension, affording users the capacity to visualize and manipulate data or objects that are integrated into their physical environment. The potential applications of this technology are numerous and diverse. The applicability of this technology in the industrial sector is discussed in [24,25]. The use of augmented reality allows for the monitoring of various technological processes, thereby enabling the tracking of parameter evolution and interaction with equipment. Consequently, real-time data is transmitted to the monitoring and control devices. Another area in which this technology can be applied is in maintenance, as users can view instructions for intervention procedures as they occur. It can be argued that augmented reality represents a fundamental technology in the context of Industry 4.0. As outlined in [26], a multitude of applications can be developed, and the constraints of this technology can be discerned. The primary concept revolves around the potential applications of augmented reality (AR) technologies in the domain of system maintenance. The fundamental principle of these augmented reality technologies is to enhance automation and information transmission. In [27], a variety of interfaces are presented that allow operators to monitor pertinent details regarding the tasks they are required to perform. The design and implementation of a system for determining the performance of technological processes are described in detail. In [28], an application that employs augmented reality glasses enables users to view instructions while engaged in assembly operations. The primary software utilized for the development of this application is Unity. An additional illustration of the application of augmented reality technologies can be observed in reference [29].

This paper presents a description of the ways in which augmented reality can be employed to facilitate interaction with a robot. Consequently, the integration of this technology facilitates the enhancement of technological processes through the incorporation of diverse methodologies, including maintenance and repairs, the development of multiple prototypes, and the secure testing of a facility. These aspects are also discussed in references [30,31]. A comparable software is presented in [32]. Therefore, the advantage of these augmented reality technologies is that technological processes can be developed and monitored with greater ease, and data can be collected in real time, enabling the user to make prompt decisions regarding any errors that may occur during operation. Furthermore, the implementation of augmented reality in training can markedly enhance the quality of staff development. This is achieved by providing employees with interactive simulations and direct visual guides during operations. Furthermore, the maintenance of equipment can be made more efficient, as technicians are able to access crucial information and step-by-step instructions directly in their field of view, thereby reducing downtime and associated costs. This results in enhanced productivity and optimized resource utilization across a range of industrial sectors.

## 3. Proposed System

**Figure 1:**
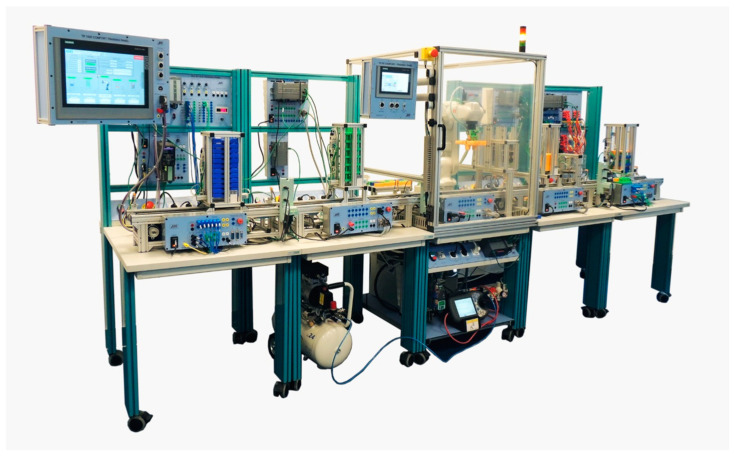
Flexible assembly line.

The methodology described below is employed to propose a solution for the acquisition and monitoring of data in the context of Industry 4.0. This is exemplified by the implementation of a method that enables the monitoring of the operation of an industrial assembly line. The assembly line comprises five stations that collectively undertake the assembly of a 3D-printed part. Figure 1 depicts the configuration of the assembly line.

**Figure 2:**
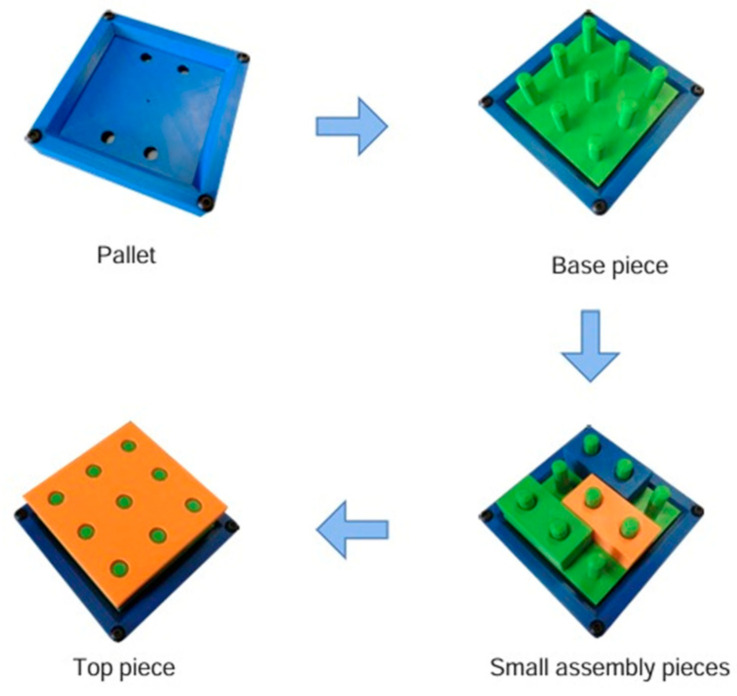
The steps of assembling a piece.

As illustrated in Figure 1, the industrial line comprises a series of machinery and apparatus through which a three-dimensional component can be assembled. The aforementioned part is composed of a multitude of constituent elements, which are themselves smaller components. Each component of the final product is produced by a designated station. The transfer of components from one station to another is facilitated by the use of conveyor belts. Subsequently, the assembled piece is stored at the final station. Figure 2 depicts the configuration of the assembled part produced via this industrial line.

**Figure 3:**
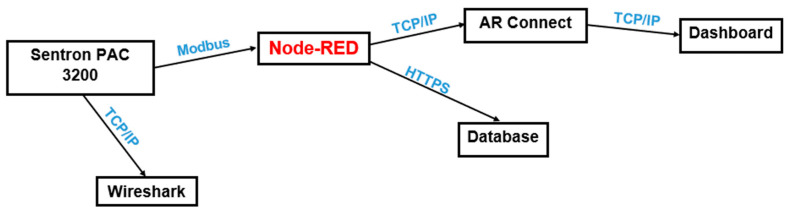
The architecture of the system.

The system designed to monitor this industrial assembly line is composed of multiple hardware and software elements, through which operational data can be accessed and transmitted. The Node-RED 3.1 interface represents the primary component of the proposed system’s architectural design. The Node-RED 3.1 interface employs a variety of nodes for the management and processing of data. Figure 3 depicts the proposed system’s architectural design.

A network analyzer, specifically the Sentron PAC 3200 2.1.0, was employed to gather data pertaining to energy consumption. This is accomplished through the use of the Modbus TCP communication protocol, which is specific to devices that are connected via Ethernet. The server side is represented by the network analyzer, while the client side is represented by a control panel. The data is then interfaced through a control panel created with Node-RED 3.1 libraries by calling multiple nodes. The control panel is represented visually through the use of augmented reality glasses. The aforementioned data are accessed via an application developed in Unity, which accesses the Node-RED 3.1 page link, thereby enabling data visualization through augmented reality. Subsequent analysis of the data is conducted using a MySQL database.

Communication monitoring and troubleshooting of potential errors that may arise during operation are conducted using the Wireshark software 4.2.1. In addition to identifying communication errors, this software is capable of determining the IP addresses of the components utilized in this application. The contribution of this work is the establishment of a novel connection between the Sentron PAC 3200 2.1.0 network analyzer and augmented reality, utilizing the Modbus TCP and HTTPS communication protocols.

A variety of hardware and software resources were employed in the course of this project. The hardware resources employed in this project include the HoloLens augmented reality glasses and the Sentron PAC 3200 2.1.0 network analyzer. These elements facilitate the monitoring of fundamental electrical network parameters, including voltage, electric current, active power, reactive power, and power factor. All of the aforementioned data are interfaced using the Node-RED 3.1 software. Node-RED 3.1 is an open-source platform that facilitates the development of a range of Internet of Things (IoT) applications. It provides a graphical interface for connecting disparate devices, thereby facilitating the integration of multiple technologies and communication protocols.

Another open-source component utilized is the MySQL database, which is employed for the storage and analysis of data pertaining to the operation of the industrial assembly line and the impact of equipment operation on the data state. The Unity software is employed for the creation of the application that serves as the interface between the control panel and augmented reality. The Wireshark software 4.2.1 facilitates communication between devices. This tool is employed for the analysis of the network, whereby screenshots of the network traffic are captured and a detailed analysis of the interactions between devices, system security, and the communication protocols used is permitted.

**Figure 4:**
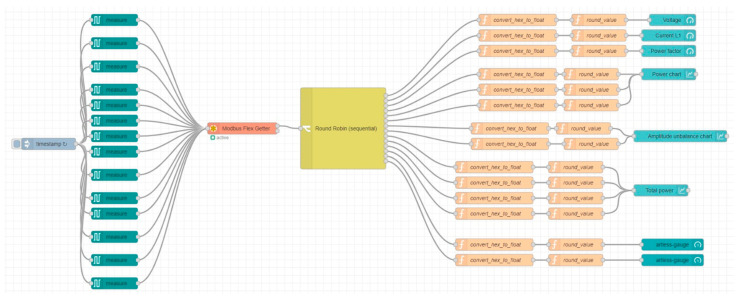
Node-RED 3.1 interface.

For the successful completion of this project, it is essential to define and configure the components involved correctly. This stage is of paramount importance to guarantee the optimal functioning of the entire system and to fulfill the project’s objectives. It is essential to configure and parameterize the equipment in order to eliminate potential errors and ensure compatibility between elements. It is crucial to adhere to the project’s specific requirements and modify the parameters as necessary to attain the desired outcome. The Modbus TCP protocol is employed for the transmission of data. Figure 4 illustrates the structure of the Node-RED 3.1 interface and the nodes utilized for data transmission and processing.

In the context of data processing, a range of functions are employed. The principal component is the “measure” block. In this block, the apparatus utilized for the measurement of specific characteristics is selected, as well as the characteristics themselves. The Sentron PAC 3200 2.1.0 device transmits a range of data pertaining to the energy consumption of the industrial installation. The data is transmitted as two values, represented in hexadecimal format, at an interval of one second. The “inject” block guarantees constant data transmission. This block allows the user to set the data transfer rate. The subsequent “measure” blocks are employed for the selection of the parameters to be monitored. In order to establish a connection with the Modbus server, the “Modbus flex getter” block is employed. In this node, the equipment’s IP address, communication protocol, and communication port are configured for the purpose of data monitoring.

The subsequent element is a node designated “Round Robin”, which ensures the uniform distribution of data. This node employs a data transfer optimization function. The objective is to reduce the overall volume of data transmitted across all channels, as outlined below:

(1) $$f\left(\gamma \right)=\propto \sum \gamma_{i}\cdot d_{i}^{2}+\beta \sum p_{i}^{2}$$

(2) $$\sum d_{i}^{2}\leq D$$

(3) $$\sum \gamma_{i}=1$$

(4) $$\sum p_{i}\leq P_{imp}$$

1 ≤ *i* ≤ *N* (5)

*D*—total channel
*γ_i_*—optimization variable
*d_i_*—communication channel
*p_i_*—data losses
∝, *β*—optimization coefficients
*N*—the total number of components or channels

**Figure 5:**
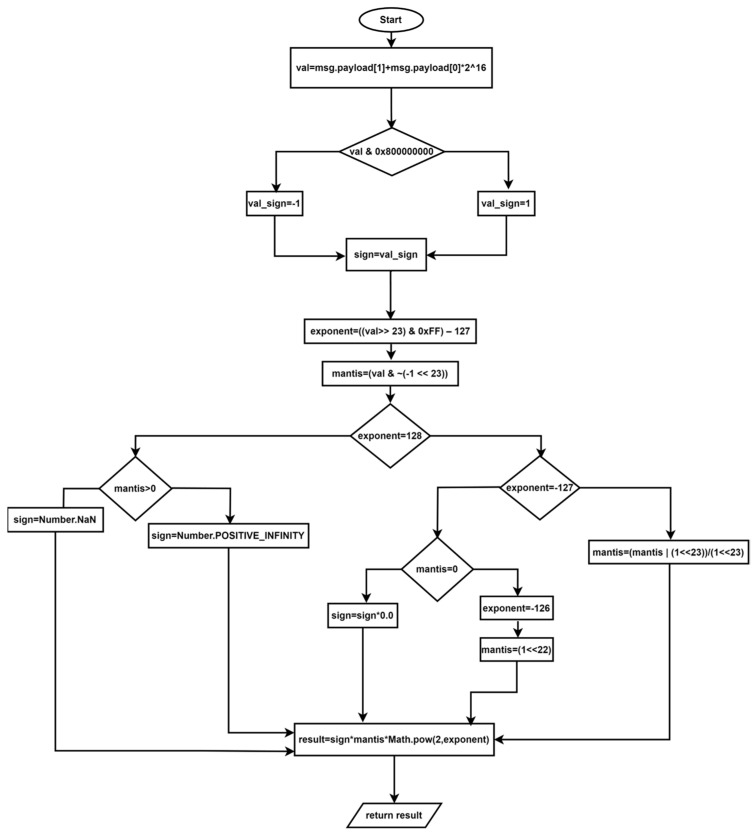
Flowchart for data conversion algorithm.

Following distribution, the data is subjected to two functions, the objective of which is to convert its hexadecimal value and estimate it. It is necessary to perform mathematical operations in order to convert the data from base 16 into a “float” format. The initial operation entails the extraction of the values obtained from the network analyzer. The resulting values are then concatenated to form a 32-bit value. Subsequently, the resulting value is converted from hexadecimal to “float” format through the utilization of a dedicated function within the Node-RED 3.1 development environment. The subsequent step is to eliminate the sign bit and the exponent portion, leaving solely the mantissa. To facilitate comprehension of this algorithm, Figure 5 presents a flowchart, and the algorithm below delineates the operations performed to achieve the data conversion process.

 val1←msg.payload[1]
 val2←msg.payload[0]
 Mot32←val1+val2*65536
 sign←(Mot32 & 0 × 800,000,000)? −1:1
 exponent←((Mot32 >> 23) & 0 × FF) − 127
 number←(Mot32 & ~(−1 << 23))
  **IF** exponent==128 **THEN**
    sign←((number)? Number.NaN:Number.POSITIVE_INFINITY)
  **END_IF**
  **IF** exponent==−127 **THEN**
    **IF** number==0 **THEN**
        signal←signal*0.0
    **END_IF**
    exponent←-126
    number←(1 << 22)
  **END_IF**
 msg← sign∗number∗Math.pow(2,exponent)
**return** msg

In order to implement the aforementioned algorithm, the JavaScript programming language is utilized. To specify the number of decimal places in the result of the data conversion function, an implemented function is utilized that employs the method msg.payload.toFixed. The code snippet allows the user to select the number of decimal places to be included with the obtained values. Subsequently, the obtained and processed data are represented using several predefined virtual tools. In regard to the communication between elements, a variety of communication protocols are employed. The primary communication protocol employed for the purpose of collecting data from the network analyzer is the Modbus TCP protocol. This represents a client/server communication protocol. In the developed application, the server is represented by the Sentron PAC 3200 2.1.0 network analyzer, while the client is the interface developed in Node-RED 3.1.

**Figure 6:**
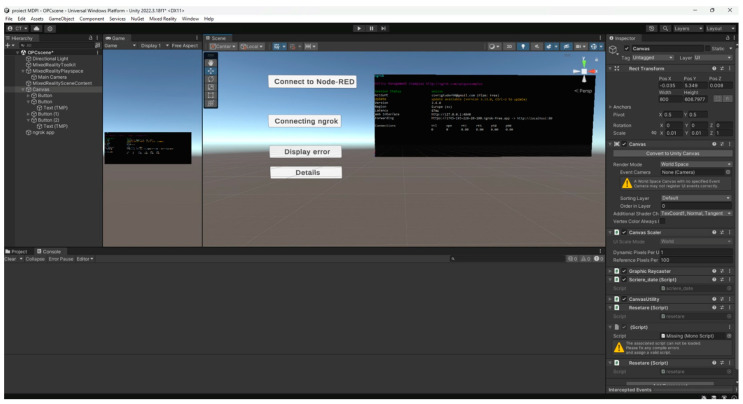
Unity interface.

In order to access the aforementioned interface via augmented reality glasses, an application developed in Unity is utilized. The application contains three buttons that facilitate the following functions: establishing a connection between the application and the Node-RED 3.1 interface to retrieve data, establishing a connection between the application and the Ngrok server to make the Node-RED 3.1 interface accessible through augmented reality, and displaying any communication errors. The application allows users to remotely access operational data from an external internet network. Consequently, remote monitoring of data is feasible. Figure 6 illustrates the structure of the utilized application.

## 4. System Implementation

This work presents the implementation of a system that enables the monitoring of operational parameters and energy consumption of an assembly line. The dashboard provides users with real-time information regarding the status of the equipment. The dashboard enables users to monitor the progression of parameters, equipment status, and any operational irregularities. The data is automatically obtained through the implementation of algorithms within the augmented reality framework, and the results generated by these algorithms are subsequently displayed through the dashboard.

The use of augmented reality (AR) represents a novel approach to the collection and integration of data pertinent to the operation of an industrial assembly line, offering a number of notable benefits. This technology enables the integration of virtual and physical components, allowing users to interact with both. These technologies facilitate the acquisition of real-time data regarding the operational status of equipment, as well as physical interaction with the equipment in question.

**Figure 7:**
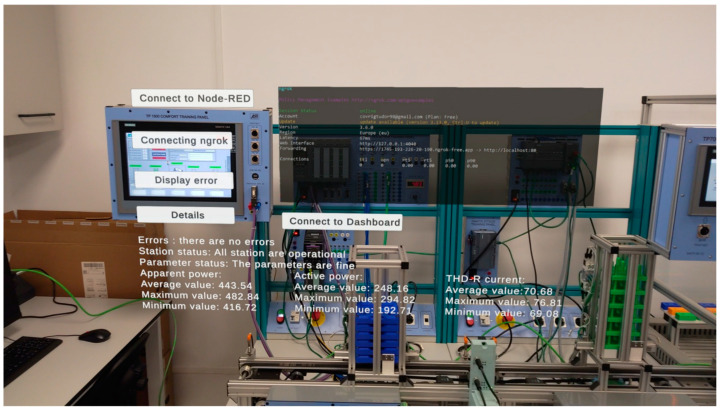
Monitoring using augmented reality.

**Figure 8:**
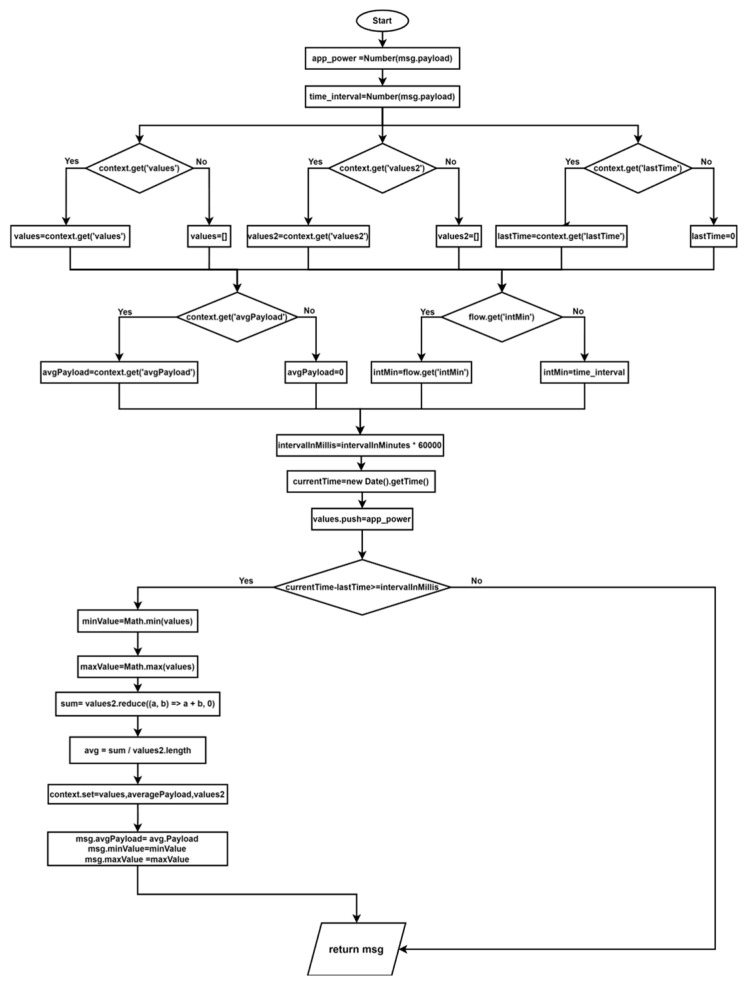
Flowchart of the methodology for operational data analysis.

As illustrated in Figure 7, the application developed with augmented reality enables the user to monitor the status of the parameters, their functionality, and any potential anomalies that may arise during operation. The data pertaining to the analyzed parameters encompass mean, maximum, and minimum values. To facilitate comprehension of this algorithm, Figure 8 presents a flowchart, and the algorithm below delineates the methodology for analyzing operational data.

app_power**←**Number(msg.payload)

time_interval**←**Number(msg.payload)

values**←**context.get(‘values’) || [ ]

values2**←**context.get(‘values2’) || [ ]

lastCalculationTime**←**context.get(‘lastCalculationTime’) || 0;

averagePayload**←**context.get(‘averagePayload’) || 0;

intervalInMinutes**←**flow.get(‘intervalInMinutes’) || time_interval;

intervalInMillis**←**intervalInMinutes * 60,000;

values.push(app_power);

**var** currentTime**←**new Date().getTime();

**if**    currentTime - lastCalculationTime >= intervalInMillis **then**

var maxValue**←**Math.max(values);

var minValue**←**Math.min(values);

var sum**←**values2.reduce((a, b) => a + b, 0);

var average = sum/values2.length;

averagePayload**←**average;

context.set**←**(‘values’, [ ]);

context.set**←**(‘averagePayload’, averagePayload);

context.set**←**(‘values2’, [ ]);

context.set**←**(‘lastCalculationTime’, currentTime);

msg.maxValue**←**maxValue;

msg.minValue**←**minValue;

msg.averagePayload**←**averagePayload;

**end_if**

**return** msg**;**

After applying the algorithm, the following data were obtained:

Apparent Power:

- Average value: 443.54
- Maximum value: 482.84
- Minimum value: 416.72

Active Power:

- Average value: 248.16
- Maximum value: 294.82
- Minimum value: 192.71

Current THD-R:

- Average value: 70.68
- Maximum value: 76.81
- Minimum value: 69.08

The JavaScript programming language was employed in the construction of this algorithm. The operation of this algorithm is as follows: at each data reading cycle from the network analyzer, the read values are stored in a list, and the current execution time is recorded in a variable. Once the allotted time has elapsed, the maximum, minimum, and average values of the parameters are calculated. Subsequently, the resulting data are transmitted to the data stream via the msg object. The algorithm is employed for the purposes of monitoring and analyzing data, thereby facilitating the observation of trends and parameter behavior within an automation system. In the event of a power outage, the discrepancy between the typical mean value and an anomalous mean value resulting from a disruption can be discerned.

**Figure 9:**
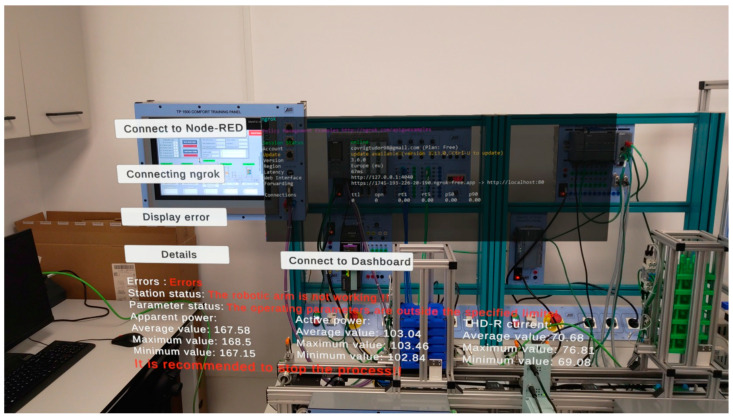
Simulation of operational errors in the assembly line.

Figure 7 depicts the optimal operational state of the assembly line, wherein all five stations are operational, and the parameters are within normal limits. To simulate operational errors, the station containing the robotic arm is disconnected from the system. These aspects are illustrated in Figure 9.

**Figure 10:**
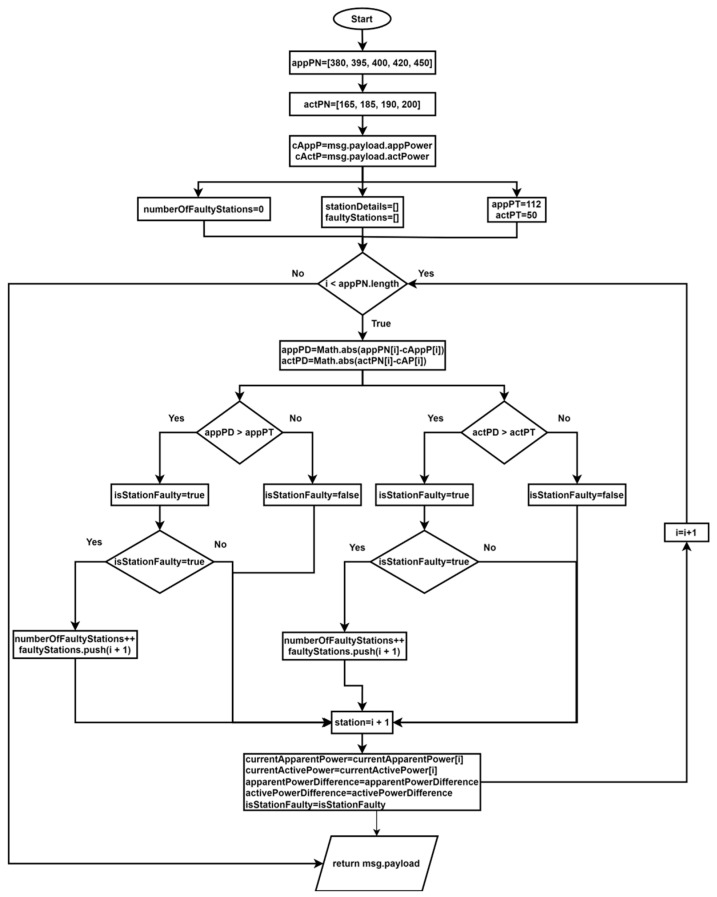
Flowchart for operational status monitoring and diagnostics.

As illustrated in Figure 9, in the event of a malfunction or deactivation of one of the stations, an algorithm is triggered to display pertinent information regarding the station’s operational status, parameter values, identification of defective stations, and recommendations for error correction. To facilitate a clearer understanding of this algorithm, Figure 10 presents a flowchart that illustrates the design of the method and the algorithm implemented using the JavaScript programming language.

**const** appPN**←**[380, 395, 400, 420, 450];
**const** actPN**←**[165, 185, 190, 200];
**const** appPT**←**112;
**const** actPT**←**50;
**let** cAppP**←**msg.payload.appPower;
**let** cActP**←**msg.payload.actPower;
**let** numberOfFaultyStations**←**0;
**let** stationDetails**←**[];
**let** faultyStations**←**[];
**for** (let i**←**0; i < appPN.length; i++) **then**
**let** appPD**←**Math.abs(appPN[i] - cAppP[i]);
**let** actPD**←**Math.abs(actPN[i] - cAP[i]);
**let** isStationFaulty**←**appPD > appPT ||actPD > actPT;
**if** (isStationFaulty) **then**
      numberOfFaultyStations++;
      faultyStations.push(i + 1);
**end_if**
stationDetails.push{
station←i + 1,
currentApparentPower←currentApparentPower[i],
currentActivePower←currentActivePower[i],
apparentPowerDifference←apparentPowerDifference,
activePowerDifference←activePowerDifference,
isStationFaulty←isStationFaulty
}
**End_for**
**IF** msg.payload **then**
    numberOfFaultyStations**←**numberOfFaultyStations,
    allStationsFunctional**←**numberOfFaultyStations**←**0,
    faultyStations**←**faultyStations,
    stationDetails**←**stationDetails
**end_if**
return msg;

The objective of the devised algorithm is to oversee the operational status of an industrial assembly line comprising five stations, employing parameters such as apparent power and active power. Initially, typical values for each of these measurements for each station are established, as well as acceptable tolerance thresholds to indicate a station’s functionality. The input message contains a payload object comprising the current values of apparent power and active power for each station. The algorithm then compares these current values with the predefined normal values. Should the discrepancy between the current measurement and the specified threshold exceed the predefined tolerance, the station is deemed to be non-functional.

The discrepancies are calculated for each station, and the algorithm determines whether a defect is present. The result includes the total number of defective stations, a Boolean indicator specifying whether all stations are functioning correctly, a list of indices of defective stations, and complete details for each station. The aforementioned information is returned in the payload object of the output message.

**Figure 11:**
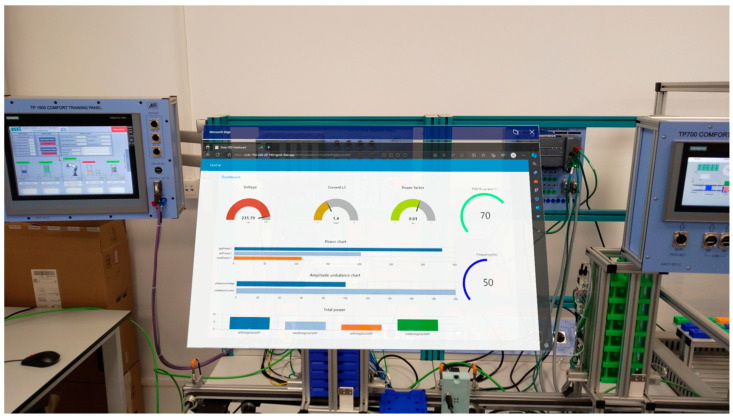
Dashboard augmented reality.

In addition to error handling and parameter status, users have the option of accessing a dashboard created with the assistance of the Node-RED 3.1 interface. The dashboard is linked to augmented reality via the nginx server. Figure 11 illustrates the structure in question.

The developed application enables the determination of the state of the equipment, energy consumption, and the impact of the equipment’s functionality on the parameters’ condition. The developed virtual tools facilitate the interfacing of values such as voltage, current, power factor, and frequency. The three graphs present a comparative analysis of active power, reactive power, apparent power, and amplitude demodulation. All obtained data can be monitored in real time, thereby enabling observation of the industrial process and the manner in which its stages influence the parameters’ condition. The data may be stored in a database for subsequent analysis, dissemination, or further processing, with the objective of facilitating decision-making and enhancing production efficiency.

Consequently, the interface furnishes the user with real-time data and enables interaction with the actual equipment. Another advantage of the application is its capacity to facilitate remote data access. This enables the user to monitor multiple technological processes in a secure manner via remote access. This method can be applied in industrial fields such as the chemical, nuclear, and metallurgical industries, thereby eliminating the exposure of supervisors to life-threatening factors. The dashboard has been designed and implemented using JavaScript within the Node-RED 3.1 interface, thereby ensuring flexibility and interactivity in the development of the interface. The dashboard was constructed in accordance with the tenets espoused by SCADA systems, employing graphical data visualization and virtual tools. A variety of objects created with JavaScript were employed to facilitate the interfacing of the data.

## 5. Results

**Figure 12:**
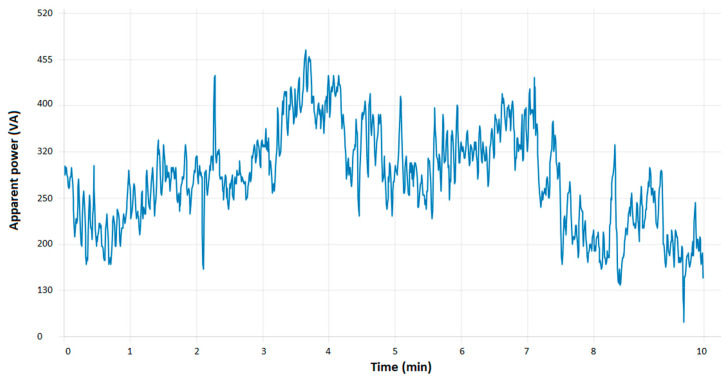
Evolution of power flow.

The application allows for the observation and monitoring of the operating parameters of the assembly line. These parameters pertain to energy consumption and its impact on the industrial process. The data obtained can be used to create a variety of charts for the purpose of analyzing the operation of the industrial assembly line. Figure 12 illustrates a chart that enables monitoring of the aforementioned parameters.

Figure 12 illustrates the temporal evolution of apparent power. From an observational standpoint, the graph illustrates that the values of the monitored parameter can fluctuate in accordance with the active process and its current operational stage. Conversely, the energy consumption is observed to be higher at certain stages and lower at others. It can thus be observed that the values of apparent power are higher when energy consumption is increased, which suggests that certain conditions and operations within the process require more energy. The graph may be utilized to identify potential sources of inefficiency within the process.

**Figure 13:**
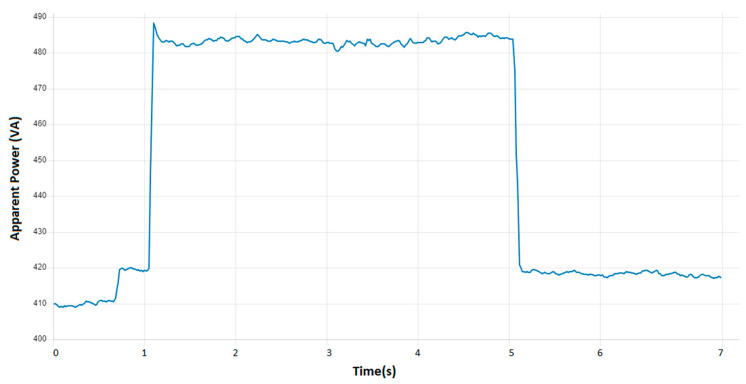
Apparent power evolution in VA during full load start process.

At the outset of the technological installation, a considerable amount of energy is consumed. The elevated energy consumption can be attributed to the necessity for greater energy expenditure by the automation equipment during the startup phase until such time as the requisite parameters have been successfully configured. Once the parameters have been configured, their state will stabilize. As the parameters are configured and stabilized, a reduction in energy consumption can be observed as the equipment adjusts its operation to meet the specific requirements of the process. Once the process has reached its normal operating stage, the apparent power may remain relatively constant, with minor fluctuations contingent upon the current stage. This phenomenon can be observed in Figure 13.

**Figure 14:**
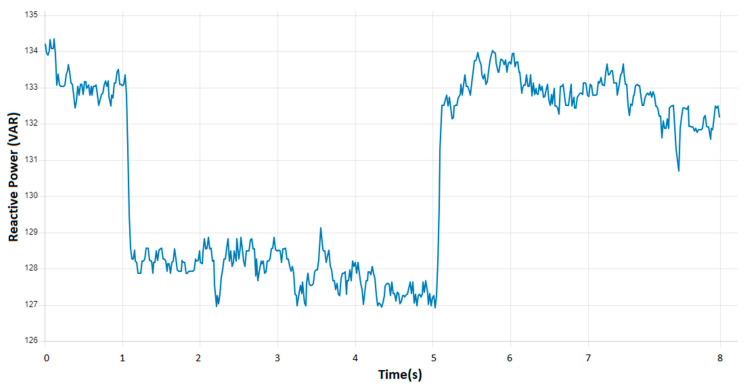
Monitoring reactive power in VAR in case of varying load due to a transient.

Another area of interest that can be investigated through the use of these charts is the impact of shutting down and then restarting the technological installation on the state of the parameters. Such an occurrence may be attributed to a power outage. The analysis of the charts can facilitate the identification of insights into the installation’s behavior during critical moments, thereby enabling the optimization of restart procedures. Furthermore, the charts can be utilized to monitor specific critical points that may arise and to ascertain which equipment necessitates a greater energy input for restarting. Consequently, the aforementioned charts facilitate the analysis of energy consumption in diverse situations and scenarios. Another crucial area of investigation is the time required for the parameters to reach a state of equilibrium. Figure 14 illustrates the temporal evolution of the parameters and their associated effects.

Furthermore, data transmission rate monitoring has been implemented in addition to the monitoring of the data itself and the effects that it undergoes. To achieve this, a node from the Node-RED 3.1 interface is employed to generate data pertaining to the number of data packets transmitted and the rate at which these packets were transferred.

**Figure 15:**
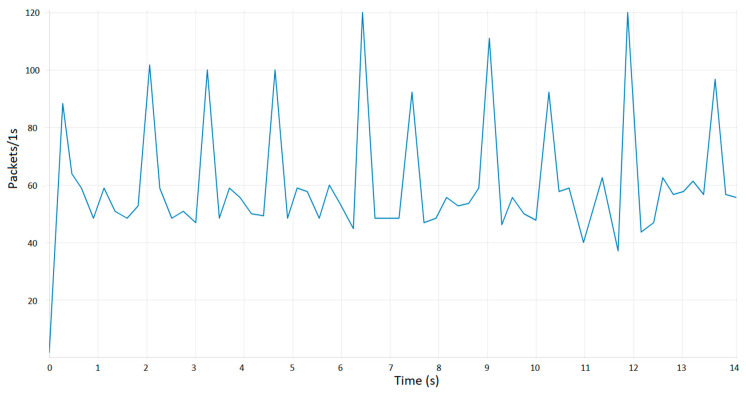
Channel optimized bandwidth utilization.

As illustrated in Figure 15, the data transfer rate exhibits fluctuating values, with the number of packets varying in accordance with alterations in the measured parameters. The Node-RED 3.1 interface functional block responsible for configuring the Modbus server is configured to initiate data transfer only when there are changes in the parameters. Consequently, when specific apparatus within the industrial configuration is activated, the states of the parameters undergo a transformation. As illustrated in the graph, an increase in energy consumption by the equipment results in a notable rise in the number of packets exchanged between the client and server. The graph enables the observation of fluctuations in the values and states of the parameters. Another aspect of the application that is monitored is its resource consumption. By monitoring these parameters, it is possible to identify where the application is requiring additional resources and to ascertain whether further resources are necessary. The subsequent figure illustrates the application parameters during operation.

**Figure 16:**
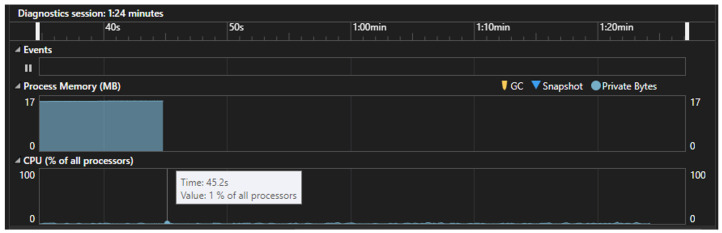
Operating parameters of the application.

Figure 16 shows the resource consumption of the application. According to this, 479, the application uses 17 MB of memory and 1% of the CPU. Therefore, the application is 480 quite efficient, requiring minimal resources to run.

## 6. Discussion

The objective of this study is to present the results of methods for monitoring the operational parameters of an industrial line using augmented reality. The aforementioned methods permit real-time supervision of the functional parameters of the industrial line, automated data analysis, and the identification of potential operational errors. The findings of this study facilitate user interaction with industrial equipment and enhance decision-making processes by providing visual, intuitive, and readily interpretable information.

The implementation of these technologies enables the effective prevention of faults and the optimization of maintenance processes. The advantages of this research include the integration of diverse technologies and the establishment of a sophisticated system capable of displaying real-time parameter status. The system is capable of determining the status of the equipment and providing instructions to the user based on the algorithms that it has been programmed with. The limitations of this approach include the potential for real-time data transmission to be vulnerable to interception, as well as the necessity for a stable connection to data collected from the assembly line, which in turn affects the performance of the augmented reality system. The findings of this study align with those of other studies in this field, such as the study in [33], which presents a system that provides instructions for using industrial equipment, remote technical support, and error analysis. A comparable system is illustrated in [34], which oversees a manufacturing process through the utilization of assorted open-source technologies, thereby facilitating real-time data transmission. The system permits remote monitoring of the process, with the status of its parameters displayed. Another remote monitoring and control solution is presented in reference [35]. The system’s fundamental component is the Internet of Things (IoT) technology, which enables remote data access. The objective of the developed system is to enhance the remote management and monitoring of equipment. Similarly, the system described in [36] enables communication between different pieces of equipment. The system is capable of performing predictive maintenance based on the data it collects. The deployment of diverse technological solutions for the monitoring of operational processes offers a number of benefits, including enhanced operational efficiency and a reduction in the time required for troubleshooting. These technologies facilitate the automation of diagnostic processes and the optimization of maintenance procedures [37].

## 7. Conclusions

This paper presents the development of an application designed to monitor the status and operational parameters of an industrial production line. The interface enables the transmission of real-time data from devices that monitor the operational data of the industrial plant. The data acquisition process is conducted via the Modbus communication protocol. Node-RED 3.1 software is employed to construct a server that facilitates access to the aforementioned data, thereby enabling communication between the Sentron PAC 3200 2.1.0 power analyzer and the data display interface. The data may be stored in a database for subsequent analysis. The application is augmented with the use of reality technology, which facilitates enhanced user interaction with virtual objects and enables the observation of the physical process simultaneously. Communication between Node-RED, which develops the parameter analysis interface, and the monitoring system is facilitated by the Nginx server, which provides access to the aforementioned interface for the purposes of monitoring and supervising the technological process. The technology offers a multitude of advantages, enabling the user to interact with virtual objects while simultaneously observing the physical process and viewing a plethora of information related to the industrial installation. The manner in which the installation operates and the impact of the simulated process on its parameters are illustrated through the use of graphical representations of the operating parameters. The aforementioned interface thus provides an efficient means of monitoring and collecting data on the operation of the industrial plant. The system generates a variety of graphs that summarize the performance and efficiency of the industrial line, providing essential information for optimizing the system. Furthermore, the application can be accessed remotely, enabling users to retrieve data from any location.

A future development of this project will entail the introduction of control over the industrial assembly process. Another idea is predictive maintenance, whereby various algorithms are employed to predict failures or maintenance needs based on data related to the operation of the technological process.

## Data Availability

No new data were created or analyzed in this study. Data sharing is not applicable to this article.
